# Immune thrombocytopenic purpura presenting in a patient after renal transplant for diabetic nephropathy

**DOI:** 10.1186/s12882-018-0868-7

**Published:** 2018-03-20

**Authors:** Raja Muhammad Rashid, Zahid Nabi, Ahmad Zaki Ansari, Quratul-ain Qaiser

**Affiliations:** 1 Department of Nephrology, KRL General Hospital, Islamabad, Pakistan; 2 Rawalpindi Medical College, Rawalpindi, Pakistan

**Keywords:** Renal transplant, Immune thrombocytopenic purpura (ITP), Immunosuppression, Post-transplant thrombocytopenia

## Abstract

**Background:**

Immune thrombocytopenic purpura (ITP) is primarily characterized by immune-mediated destruction of platelets in circulation. Major treatment options range from careful observation, steroids, immunosuppressive medications, immunoglobulins to splenectomy. Interestingly and rarely, ITP has also been reported after solid organ transplantation in patients receiving immunosuppressive medications. While the incidence of new onset ITP after solid organ transplant is comparatively well documented, new onset ITP after renal transplant has only been reported in two patients. Both these patients underwent renal transplant for underlying Immunoglobulin-A (IgA) nephropathy and were treated effectively with steroids. We present successful management of the first reported case of new-onset ITP presenting after renal transplant in a patient with underlying diabetic nephropathy. The case report discusses the potential management strategies in such a novel scenario aiming simultaneously for a well-functioning renal graft, adequate hemostasis, minimum therapy- related morbidity and least cost implications for the patient.

**Case Presentation:**

A 43-year-old male with hypertension and diabetes mellitus (DM), complicated by nephropathy and retinopathy, underwent pre-emptive living related renal transplant by donation from his 33-year-old wife. His immediate post-transplant period was unremarkable. Six months after the transplant, he presented with isolated thrombocytopenia. An extensive workup revealed no clinical or laboratory evidence of unusual substance intake, infection, hemolysis, microangiopathy, autoimmune disease or hematological malignancy. Eight months after the transplant, while the patient was maintained on steroids, cellcept and tacrolimus, his platelet count dipped to 13,000/microL and he had an episode of mild epistaxis. He was administered steroids in line with the adult ITP management protocol. Steroids were well tolerated, and platelet counts showed a good response to therapy. Steroids were then successfully tapered over the next ten weeks with steady and acceptable platelet counts and graft function.

**Conclusions:**

The case report discusses the diagnostic considerations and successful management of new-onset post-renal transplant ITP. It also highlights the various therapeutic options available in the medical armamentarium including shuffling of immunosuppressive drugs, rituximab, thrombopoietin receptor agonists (TPO’s) and splenectomy for their potential use in complicated scenarios like relapsing, or steroid-refractory post renal transplant ITP.

## Background

Immune thrombocytopenic purpura (ITP) is primarily characterized by immunologically mediated destruction of platelets in circulation at a rate surpassing production by compensating bone marrow [1]. In adults, the incidence is variably reported to be 22 to 39 individuals per million per year and prevalence being 50 to 100 individuals per million per year in USA and Europe respectively [2]. Clinical presentation varies between asymptomatic isolated thrombocytopenia, mucocutaneous bleeding and bruising. There is no gold standard for diagnosis. However, isolated thrombocytopenia, normal peripheral smear, with exclusion of apparent conditions and drugs, helps in the clinical diagnosis [1] Treatment options range from careful observation steroid administration, immunosuppressive drug treatment to splenectomy. Infrequently, ITP has also been reported after solid organ transplantation in patients already on immunosuppressive drugs [2–5]. Here we present the case of a middle- aged man with diabetic nephropathy who underwent living related renal transplant and developed isolated thrombocytopenia six months later. After extensive workup, he was successfully treated along the lines of new- onset ITP post renal transplant.

## Case Presentation

A 43-year-old male with hypertension and diabetes mellitus (DM), complicated by diabetic nephropathy and retinopathy, underwent preemptive living related renal transplant by donation from his 33-year-old wife on 6th of May 2015. Neither the donor, nor the recipient had any history of autoimmune diseases or blood dyscrasias and both were IgG seropositive for Cytomegalovirus (CMV) and Epstein–Barr virus (EBV). Prior to transplantation, there was no history of blood or blood product transfusion. HLA tissue typing revealed antigen match at loci A*03, B*57, DRB1*03 and DQB1*05. The patient received Basiliximab at induction as per protocol and was maintained on a triple immunosuppressive regimen of tacrolimus, mycophenolate and steroids. Tacrolimus trough levels were successfully maintained between 7 to 12 ng/ml and his immediate post-transplant period was uneventful. Steroids were tapered over a period of 8 weeks to a dose of 5 mg once daily. He received standard prophylaxis with valganciclovir, nystatin oral drops and trimethoprim-sulfamethoxazole.

Six months post-transplant, upon follow- up, there was no history of any preceding or current fever, flu- like illness, cough, dyspepsia, gastrointestinal discomfort, altered bowel habits, rash, skin discoloration, body aches or joint pains. He denied taking any non-prescribed drugs including herbal, homeopathic or traditional medications. His blood sugars were adequately controlled. The examination was unremarkable for petechiae, bruises, edema, lymphadenopathy, hepatomegaly or splenomegaly. Laboratory reports were normal except for isolated thrombocytopenia with a platelet count of 40, 000/microL (40, 000/microL, 40 × 10^9^/L, and 40,000 × 10^9^/mL). Blood work showed a normal white blood cell count (9.2 × 10^3^/microL), normal hemoglobin level (14.5 g/dl) and an absence of sinister cells or fragmentation in the peripheral smear. His renal function tests (creatinine 1.2 mg/dl), liver function tests, and electrolytes were normal.

After reconfirming the low platelet count in citrated sample, extensive work- up was carried out to ascertain the cause. C - reactive protein (CRP) was within limits and blood and urine cultures showed no growth. Viral serologies and PCR based detection was negative for hepatitis B, hepatitis C, CMV and EBV. Serological workup was negative for Human Immunodeficiency Virus (HIV) and Brucella. Malaria was tested negative by smear and ICT method. Serology and stool antigen tests were negative for *H. pylori*. Autoimmune workup including ESR, antinuclear antibody (ANA), anti-double stranded DNA antibody (anti- dsDNA), extractable nuclear antigens profile (ENA profile), Coomb’s test (DAT) and lactate dehydrogenase (LDH) levels were normal. The patient was euthyroid clinically and biochemically. Abdominal imaging was also normal.

**Fig. 1:**
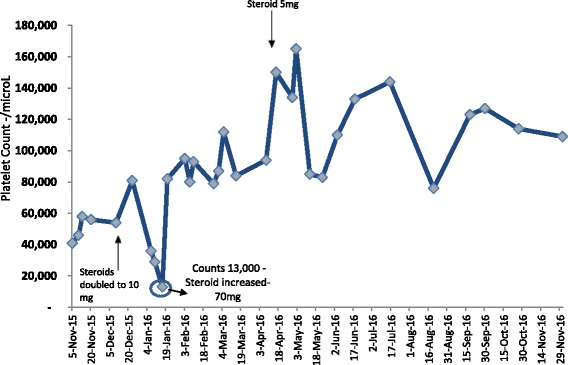
Platelet counts over time and response to treatment. Normal platelet counts are taken to be 150–450 × 10^3^/microL. (SI units 150–450 × 10^9^/L). Hemoglobin, RFT’s and LFT’s remained normal throughout this period

The drug chart was reviewed, and the dose of mycophenolate was reduced from 1 g/day to 500 mg/day without any effect on platelet counts, which remained within 40,000/microL (40× × 10^9^/L) to 50,000/microL (50 × 10^9^/L). While the bone marrow biopsy was being arranged, the dose of prednisolone was increased from 5 mg/day to 10 mg/day on December 10, 2015. Platelets showed a subsequent transient increase from 54,000/microL to 81,000/microL. In liaison with the hematologist, bone marrow biopsy was performed on December 23, 2015. It was negative for any infiltrative lesions and was consistent with peripheral destruction. In the month of January, while patient was still being maintained on 10 mg prednisolone, his platelets showed a progressive downward trend. While all other blood indices and chemistry parameters remained normal, he was re- admitted with platelet count of 29,000/microL. On January 16, 2016, his platelet counts dropped to 13,000/microL and he had an episode of mild epistaxis. 250 mg of pulse methylprednisolone was administered on Day 1 followed by 100 mg on Day 2. His platelet counts jumped to 48,000/microL on Day 3 and 80,000/microL on Day 5. His oral prednisolone was subsequently increased to 70 mg per day and TMP-SMX prophylaxis was reinstituted. The platelet counts showed adequate response and were within safe limits within 2 weeks. His blood sugars were well controlled with up- titration of both basal and bolus insulin. Steroids were gradually tapered over the next 10 weeks during which platelet counts remained steady and within safe limits (ranging from 90,000/microL to 180,000/microL). Mycophenolate was increased to 1 g per day upon tapering of the steroids. The patient was maintained on prednisolone 5 mg daily with excellent graft function and acceptable platelet counts for almost an entire year of follow-up (Fig. 1).

## Discussion and conclusion

Barring isolated thrombocytopenia, post-renal transplant cytopenias including anemias, bicytopenias and pancytopenias are relatively well studied and documented. The cause of thrombocytopenia is generally connected with suppression of other cell lines and includes immunosuppressive or antimicrobial drugs, infections, sepsis and malignancies [6]. The prevalence of thrombocytopenia < 100,000/microL (< 100 × 10^9^/L) and severe thrombocytopenia < 50,000microL(< 50 × 10^9^/L) after renal transplant, without specifying whether isolated or otherwise, was reported to be around 30% and 4% respectively in the adult Chinese population [7]. ITP in the setting of other solid organ transplants is comparatively well- reported. The incidence of new onset ITP post-orthotopic liver transplant was reported to be 0.7% in 1105 liver transplant patients over a period of 15 years at Ann Arbor University. Such cases are, however reported very rarely after renal transplant [2–5].

Including the present case, all reported cases of new onset ITP post renal transplant, have been diagnosed in middle aged men. In the general population, however, ITP is more prevalent in 30- to 60-year-old females [2, 4, 5]. Orchard TR et al. reported successful treatment of ITP with steroids in a 33-year-old patient with IgA nephropathy who developed thrombocytopenia nine months after his kidney transplant while he was taking cyclosporine, prednisolone and azathioprine [4]. Talaulikar D. et al also reported ITP in a post- renal transplant setting in a 42-year-old male which was managed effectively with steroids [5]. Our case is the first reported instance of new- onset ITP in a patient with renal transplant for diabetic nephropathy.

The diagnosis of ITP is suggested by isolated thrombocytopenia with the peripheral blood smear helping to exclude microangiopathic hemolytic anemia, thrombotic thrombocytopenic purpura, platelet clumping, and hemolysis. Clinically apparent conditions involving viral infection, medications, malignancy, systemic disease, disseminated intravascular coagulation (DIC), and sepsis need to be ruled out and excluded. Hepatitis C and Human Immunodeficiency Virus testing is recommended for all adult patients with ITP [1]. Bone marrow biopsy is not mandatory but verifies ample megakaryocyte lineages and excludes infiltrative disorders and malignancy [1, 3, 8]. Routine testing for platelet specific antibodies is still not recommended for diagnosis and management of ITP [1, 3, 9]. In refractory cases, proponents find potential utility of a positive glycoprotein antibodies test from a reference laboratory, where available, as an adjunct to the clinical diagnosis [9]. Rapid response to steroids, when observed, is also suggestive of ITP and renders alloimmune thrombocytopenia less likely. Transplant- mediated alloimmune thrombocytopenia (TMAT) is another very rare entity causing isolated thrombocytopenia. Its clinical course is, however, explosive: generally presenting in early post-transplant period and refractory to usual medical interventions [10].

While encountering cytopenias, the usual suspects in clinical practice are drugs and infections. We reviewed our patient’s chart carefully and tailored the prescription to the minimum number of drugs required. The dose of mycophenolate was reduced to half without any effect on cell indices. We emphasized strict dietary and lifestyle modifications, thereby withdrawing rosuvastatin and ranitidine without any subsequent effect on platelet counts. Our patient demonstrated no clinical signs of illness, infection or malignancy. His graft function and blood pressure remained stable. While excluding infection, it is important to keep local disease burdens in mind. Cases of thrombocytopenia have been reported with malaria, *H. pylori* and tuberculosis; which were excluded clinically and on laboratory and radiological parameters. *H. pylori* was tested negative by serology and stool antigen test. In addition to serologies, where pertinent, our work up included PCR based detection for common infections including CMV, Hepatitis B and Hepatitis C. Where clinically relevant and available, testing for Varicella zoster and Parvo-virus B19 could be considered. The donor was also evaluated, revealing normal blood indices and no evidence of infection in vaginal and urethral cultures. In periodic re- assessments during follow-up, the patient did not demonstrate any other disease indicators like anemia, leucopenia or lymph node enlargement.

The treatment of ITP in adults requires individualized comprehensive assessment of the patient’s occupation, bleeding risk, co- morbidities, therapy related side effects, cost implications and preference of the patient. Therapy is generally recommended for patients with a platelet count below 30,000/microL (< 30 × 10^9^/L). Aside from thrombopoietin receptor agonists (TPO’s), most ITP therapies inhibit active B cells, T cells and/or monocytic-macrophage system. Steroids are the mainstay of treatment and act on the immune system globally by not only functionally diminishing reactivity of T- and B-cell; but also, by inducing tolerance patterns in dendritic cells, cytokines and T cells [11]. IVIG and anti- D can also be used in special circumstances. Second line options include splenectomy, rituximab and thrombopoietin receptor agonists. Rituximab in combination with steroids has been shown to diminish B cell lines while considerably increasing regulatory T cells [11]. Individual successes have been reported, albeit inconsistently, with azathioprine, cyclosporine, mycophenolate, cyclophosphamide, vincristine and danazol. Regardless of the therapy selected, the goal is to achieve patient- specific acceptable platelet counts that are sufficient to prevent bleeding; rather than attempting to bring the counts to normal ranges. Our patient showed a good response to steroid therapy and we were able to taper them to 5 mg over the course of 10 week. Platelet counts remained above 50,000/microL (50 × 10^9^/L) throughout the course of follow-up, demonstrating sustained response to therapy. Strict lifestyle modification, dietary instructions, and close monitoring with appropriate titration of insulin kept his sugars within acceptable limits during the intensive steroid therapy phase. Since he is a driver by profession, he was counseled to switch from driving on highways to driving only on local routes, thereby minimizing the potential risk of high speed trauma. There was no resultant decline in his quality of life due to low platelet counts.

In adults with ITP, spontaneous remissions are reported in 10% of the patients, usually within the first six months. One- third to two- thirds of these patients will have persistent or chronic ITP with acceptable platelet counts either after first line therapy or after spontaneous remission [12, 13]. When treatment is needed, the longer duration of steroid therapy required in adults poses additional management challenges in relapses of new onset ITP post-transplant. Such challenges will exponentially increase if relapse is either steroid- dependent or refractory to steroids. The therapy thenceforth will have to be tailored to maintain a well-functioning renal graft, adequate hemostasis and minimum therapy- related morbidity to patient. Talaulikar D. et al. successfully treated relapse in their patient with intermediate low- dose steroids [5]. For our patient, the goal will be to keep the platelet counts above 30,000/microL (30 × 10^9^/L) with the least number of medications possible. In the case of a first relapse, steroid therapy may suffice. IVIG with low- dose steroids are another option for such patients and will work best if disease remits early in its natural course. There is anecdotal evidence from local experience that azathioprine has a better response in ITP in our population. Switching from mycophenolate to azathioprine is another consideration with the aim of modifying the immune response that may allow sustained and acceptable platelet response in our patient. For steroid dependent or steroid refractory disease in our case, splenectomy can be reserved as the last resort considering allograft medications, local infection burden in hospital and patient preference. Recent reports show good short- term and modest long-term responses in ITP with rituximab alone or in conjunction with other agents as an option to avoid splenectomy. In fact, rituximab and splenectomy have both been used with some success for treating refractory ITP in the post- liver transplant setting [2].

Uncommonly, uremia and ITP can coexist in a single patient. Patients in remission, or those with platelet counts more than 75,000/microL (75 × 10^9^/L), may choose dialysis or renal transplant with necessary adjustments. Severe thrombocytopenia, however, poses additional bleeding risks in either renal replacement modality. Isolated reports show that post-transplant immunosuppressive medication resulted in stable platelet counts in patients with ITP who underwent renal transplant [14]. Simultaneous splenectomy and renal transplant have also been carried out for steroid refractory ITP and chronic renal failure [15]. Post renal transplant ITP cases like these can help patients make informed decisions equipped with the knowledge of the possibility of persistence of ITP post-transplant; and can also help guide immunosuppressive regimens in such exceptional scenarios.

Our case report highlights successful management of ITP with steroids in the setting of renal transplant and diabetic nephropathy. It would be interesting to see how the natural course of disease differs in post renal transplant setting than in normal adults. In addition to posing management challenges, such novel cases also open avenues for translational research and advancement in understanding of disease pathology and treatment responses.

## Abbreviations

ANA: Antinuclear antibody
Anti- dsDna: Anti-double stranded DNA antibody
CMV: Cytomegalovirus
CRP: C - reactive protein
DIC: Disseminated intravascular coagulation
DM: Diabetes mellitus
EBV: Epstein–Barr virus
ENA profile: Extractable nuclear antigens profile
ESR: Erythrocyte sedimentation rate
HIV: Human immunodeficiency virus
ITP: Immune thrombocytopenic purpura
LDH: Lactate dehydrogenase
TMAT: Transplant mediated alloimmune thrombocytopenia
TMP-SMX: Trimethoprim-Sulphamethoxazole
TPO’s: Thrombopoietin receptor agonists

## References

[1] Neunert C, Lim W, Crowther M, Cohen A, Solberg L, Crowther MA (2011). The 1. American Society of Hematology 2011 evidence-based practice guideline for immune thrombocytopenia. Blood.

[2] Taylor RM, Bockenstedt P, Su GL, Marrero JA, Pellitier SM, Fontana RJ (2006). Immune thrombocytopenic purpura following liver transplantation: a case series and review of the literature. Liver Transpl.

[3] Diaz GC, Prowda J, Lo IJ (2008). Transplantation-mediated alloimmune thrombocytopenia: guidelines for utilization of thrombocytopenic donors. Liver Transpl.

[4] Orchard TR, Neild GH (1997). Immune thrombocytopenic purpura presenting in an immunosuppressed patient after renal transplantation. Nephrol Dial Transplant.

[5] Talaulikar D, Falk M, Talaulikar G, Pidcock M (2007). Immunethrombocytopenia after renal transplantation for IgA nephropathy. Acta Haematol.

[6] Yang Y, Yu B, Chen Y (2015). Blood disorders typically associated with renal transplantation. Front Cell Dev Biol.

[7] Xie L, He S, Fu L (2013). The prevalence and risk factors of thrombocytopenia after living-related renal transplantation in Chinese adult recipients. Transplant Proc.

[8] Cines DB, Blanchette VS (2002). Immune thrombocytopenic purpura. N Engl J Med.

[9] British Committee for Standards in Haematology General Haematology Task Force (2003). Guidelines for the investigation and management of idiopathic thrombocytopenic purpura in adults, children and in pregnancy. Br J Haematol.

[10] West KA, Anderson DR, McAlister VC, Hewlett TJ, Belitsky P, Smith JW (1999). Alloimmune thrombocytopenia after organ transplantation. N Engl J Med.

[11] Johnsen J (2012). Pathogenesis in immune thrombocytopenia: new insights. Hematology Am Soc Hematol Educ Program.

[12] Neylon AJ, Saunders PW, Howard MR, Proctor SJ, Taylor PR (2003). Clinically significant newly presenting autoimmune thrombocytopenic purpura in adults: a prospective study of a population-based cohort of 245 patients. Br J Haematol.

[13] Rodeghiero F, Stasi R, Gernsheimer T (2009). Standardization of terminology, definitions and outcome criteria in immune thrombocytopenic purpura of adults and children: report from an international working group. Blood.

[14] Einollahi B (2009). Renal transplantation and idiopathic thrombocytopenic purpura: two case reports. Transplant Proc.

[15] Hwang EM, Woo HY, Choi BS, Yang CW, Kim YS, Moon IS, Bang BK (2005). Renal transplantation in a patient with idiopathic thrombocytopenic purpura. Korean J Intern Med.

